# Lessons learnt from the 2021 Pacific Northwest heat dome: a qualitative study of western Washington’s healthcare community response

**DOI:** 10.1136/bmjopen-2024-089093

**Published:** 2025-04-17

**Authors:** Matias Korfmacher, Cat Hartwell, Kelly Hill, Nathaniel Matthews-Trigg, Jeremy Hess, Amruta Nori-Sarma, Gregory Wellenius, Nicole Errett

**Affiliations:** 1 Department of Environmental and Occupational Health Sciences, University of Washington, Seattle, Washington, USA; 2 Northwest Healthcare Response Network, Tukwila, Washington, USA; 3 Department of Global Health, University of Washington, Seattle, Washington, USA; 4 Department of Emergency Medicine, University of Washington, Seattle, Washington, USA; 5 Center for Climate and Health, Boston University, Boston, Massachusetts, USA; 6 Department of Environmental Health, Harvard T H Chan School of Public Health, Boston, Massachusetts, USA

**Keywords:** PUBLIC HEALTH, Climate Change, QUALITATIVE RESEARCH

## Abstract

**Abstract:**

**Objective:**

The 2021 Pacific Northwest heat dome was Washington state’s deadliest recorded weather event and presented unprecedented response challenges to the state’s health sector. Understanding the impacts of this extreme heat event (EHE) on the sector as well as the barriers to and facilitators of implementing effective heat response is critical to preparing for future events, which are happening more frequently in the region due to climate change.

**Design:**

Guided by an implementation science framework, we convened listening sessions and focus groups of the health sector in western Washington to reflect on regional response efforts.

**Setting:**

Health sector organisations in 15 counties in western Washington State, USA: Clallam, Grays Harbor, Island, Jefferson, King, Kitsap, Lewis, Mason, Pacific, Pierce, San Juan, Skagit, Snohomish, Thurston and Whatcom.

**Participants:**

A convenience sample of 109 listening group participants was recruited through the professional networks of the Northwest Healthcare Response Network, a regional healthcare coalition. 27 of the health sector professionals were recruited using purposive sampling to participate in seven focus groups organised by organisation type.

**Results:**

The co-presence of the COVID-19 pandemic, limited staff capacity, resource acquisition challenges and inadequate regional collaboration emerged as key barriers, while advanced planning, indoor cooling capabilities, adapting strategies to local needs, robust internal relationships and strong external partnerships were reported to facilitate effective response. Establishing centralised coordination ahead of heat events, making improvements to the cooling capabilities of the built environment, developing plans and policies for EHEs that have co-benefits for other events, adopting evidence-informed response strategies, institutionalising the knowledge and relationships developed through prior events and improving evaluative processes (such as developing real-time monitoring capacity) will enable more effective response to future EHEs.

**Conclusions:**

Western Washington’s health sector implemented EHE response activities that enabled essential service continuity, despite limited resources, unfamiliarity with EHEs and other systemic challenges. The recency of the heat dome presents an opportunity to incorporate lessons learnt into practice, policies, plans and built environment; these are necessary improvements ahead of future large-scale events the region may experience in the coming decades.

**STRENGTHS AND LIMITATIONS OF THIS STUDY**

- Our study applied an established implementation science framework (the Updated Consolidated Framework for Implementation Research) in a novel way by assessing barriers and facilitators of a suite of extreme heat event response activities.
- Participants included emergency management contacts for a diverse array of organisations across the health sector.
- Due to the participant pool and scheduling conflicts, several valuable perspectives in the health sector were absent or limited in focus group discussions, including facilities workers, tribal health entities and small healthcare organisations (eg, private practices).
- Some organisation scales or specific regions may be over-represented while others may be under-represented.
- The geographic focus on the Pacific Northwest, a region with both limited extreme heat event experience and robust emergency response infrastructure, may limit the generalisability of this study’s findings when applied to other regions.

## Introduction

 Extreme heat is the USA’s deadliest natural hazard.^1 2^ Extreme heat events (EHEs), commonly called heat waves, are extended periods of unusually high temperatures.^3^ Due to anthropogenic climate change, EHEs are increasing in frequency, length and intensity^4 5^; more people may be exposed to hazardous heat in the future.^6^ Prolonged exposure can cause acute heat illnesses like heat exhaustion, dehydration and heat stroke; and exacerbate chronic respiratory, cardiovascular, renal and psychiatric conditions.^7^,^9^ These adverse health impacts lead to unplanned emergency department (ED) visits and hospital admissions.^1 10^ Although EHEs pose a universal health risk, some populations face elevated risks, including elders, children, pregnant people, people with pre-existing physical or mental health conditions, low-income communities, outdoor workers, black, Indigenous and People of Color individuals with access and functional needs, individuals experiencing homelessness and socially isolated individuals.^7 11 12^

The health sector (encompassing healthcare, emergency management and public health agencies; health-related non-profits; and patient-facing healthcare providers)^13^ plays a critical role during EHEs by supporting prevention and risk communication activities, maintaining necessary resources to treat an influx of patients, communicating health impacts to at-risk individuals and providing life-sustaining services.^14^ Health-based heat messaging, cooling or resilience centre operation, and targeted outreach to at-risk populations (eg, well-being checks and personal fan or air-conditioning (AC) unit distribution) are common practices.^15^,^18^

Despite its importance in managing heat-related morbidity and mortality, the USA’s health sector has historically been underprepared for EHEs.^3 19^ EHEs have profound impacts on the health sector, including increased demand for health services due to direct and indirect injuries^20 21^; workforce, facility, power supply and equipment disruptions (eg, blackouts or brownouts) hindering service provision^1422^,^24^; and increased costs,^25^ necessitating preparedness. Given the unique impacts of EHEs, maintaining all-hazards preparedness is necessary but insufficient.^3^ Evidence-informed response strategies (eg, adopting and refining heat action plans, conducting heat-related surveillance, increasing access to cooling technology and identifying vulnerable populations) have been identified but not widely implemented.^3 14 26 27^ Furthermore, EHE definitions, activation protocols and preparedness and response activities vary, challenging coordination between jurisdictions and organisations.^16 28 29^ Compounding planning gaps, federal funding for EHE response has been limited.^28^ All prior requests for major disaster declarations under the Robert T. Stafford Disaster Relief and Emergency Assistance Act, which enables federal response coordination and financial assistance, have been denied because the ‘magnitude and severity’ of the events were determined to be insufficiently severe.^30^,^32^

The 2021 Pacific Northwest heat dome exemplifies the growing urgency to establish climate-resilient health systems able to respond to EHEs.^3^ Across British Columbia, Oregon and Washington, the event’s combined excess death toll was in at least the high hundreds.^33^,^35^ It was Washington’s deadliest recorded weather event, with approximately 159 excess injury deaths during the event the subsequent 2 weeks.^36^ The heat dome—unprecedented for a region largely unfamiliar with EHEs—lasted from approximately 25 June to 2 July 2021. Record-setting temperatures reached 120°F (48.9°C) in some parts of Washington, remaining high overnight.^37^ The heat dome would have been a 1-in-1000-year event between 1850 and 1900,^38^ but with just 2°C of global warming—predicted by mid-century—an event of similar magnitude could occur every 5–15 years.^38 39^ The heat dome had severe, cascading effects on the region’s health sector. Heat-related hospital visits in Alaska, Idaho, Oregon and Washington were 69 times higher during the event than the same date range in 2019.^40^ Residential facilities (eg, long-term care) found it challenging to regulate indoor temperatures without AC,^41 42^ as at the time Washington had one of the lowest proportions of residences with AC in the country (53%).^43^ Emergency medical systems (EMS) and healthcare providers experienced long wait times, overheated testing equipment and surges of ED patients,^44 45^ even as the COVID-19 pandemic restrictions were lessening in Washington.^46^

While prior studies have examined associations between the heat dome and ED visits,^47^ excess injury mortality^36^ and the climatic significance of the event,^44^ there has yet to be a systematic investigation of the health sector’s response activities; a need to identify pathways to improve the region’s preparedness and response capabilities remains. This study assesses barriers to and facilitators of heat preparedness and response activity implementation, as well as lessons learnt by the health sector in Washington, to inform future EHE response.

## Methods

This qualitative study leveraged listening sessions and focus groups with health sector professionals in western Washington. Transcripts from focus groups were thematically analysed using the Consolidated Framework for Implementation Research (CFIR), an implementation science framework, to identify and characterise barriers to and facilitators of heat response efforts undertaken by health sector organisations during the heat dome.^48^ Implementation science informs the adoption of evidence-based practice and research findings into healthcare and public health to improve the quality and effectiveness of health services.^48 49^ The CFIR examines determinants of implementation using constructs organised under five domains: innovation, outer setting, inner setting, individuals and implementation process.^48^ Online supplemental material 1 provides further comment on the use of the CFIR.

### Data collection

The research team and partners at the Northwest Healthcare Response Network (NWHRN), an emergency preparedness and response coalition of healthcare organisations and public health partners across western Washington, recruited participants for listening sessions through convenience sampling. Researchers conducted hour-long listening sessions in each of NWHRN’s four regularly scheduled service region meetings between 16 and 27 May 2022. Attendees consisted of emergency management contacts from NWHRN’s member organisations, who were provided background information, study goals and copies of an informed consent statement, then given time to ask questions. Participants gave verbal consent and recording permission in advance. Each listening session had between 20 and 36 participants, for a total of 109 participants. Researchers MK and CH took notes for analysis. Researchers purposively sampled listening session participants based on professional affiliation and NWHRN service region for focus group discussions. Of the 46 individuals contacted, 27 agreed to participate in a focus group. Seven focus groups of 3–6 participants each were arranged based on the organisation types represented among participants: (1) hospital-based clinicians; (2) local public health agencies; (3) a ‘miscellaneous health service’ group, including organisations serving older adults (‘ageing organisations’), an NWHRN staff member and an air ambulance provider; (4) long-term care; (5) local emergency management agencies; (6) hospital emergency management; and (7) home health, hospice and outpatient services. Focus groups occurred between 6 and 27 September 2022, and lasted 37–54 min. All participants provided informed consent to participate and be recorded. Online supplemental material 2 provides additional information about focus group recruitment and sampling.

Researchers developed semistructured facilitation guides (online supplemental material 3) based on CFIR domains (online supplemental material 3) for listening sessions and focus groups.^49 50^ MK and CH used listening session notes to develop summaries by facilitation question using the rapid qualitative analysis approach.^51 52^ Summaries were extracted into cells of a matrix corresponding to facilitation guide questions (columns) and organisation-type classifications (rows) to identify commonalities and differences across sessions. This synthesis informed focus group-specific follow-up questions. MK and CH audited each other’s work to promote qualitative confirmability.^51 53 54^

### Data analysis

Focus group recordings were professionally transcribed; researchers reviewed transcripts for accuracy and data familiarisation. The Updated CFIR framework informed the codebook for this study (online supplemental material 4).^48 55^ MK and CH co-coded focus group transcripts using NVivo software, resolving discrepancies through consensus, and used the framework method to analyse the data.^56 57^ This method organises data into a matrix of cases and codes so that themes can be identified and compared across units of analysis (focus groups).^58^ MK summarised data in an analytic memo to emphasise divergences and commonalities.^59 60^ CH reviewed the memo through an audit and feedback process informed by Lincoln and Guba’s approach to promote qualitative trustworthiness.^54^

### Patient and public involvement

None.

## Results

Focus group discussions revealed diverse roles and responsibilities, impacts, and barriers and facilitators to extreme heat preparedness and response.

**Figure 1:**
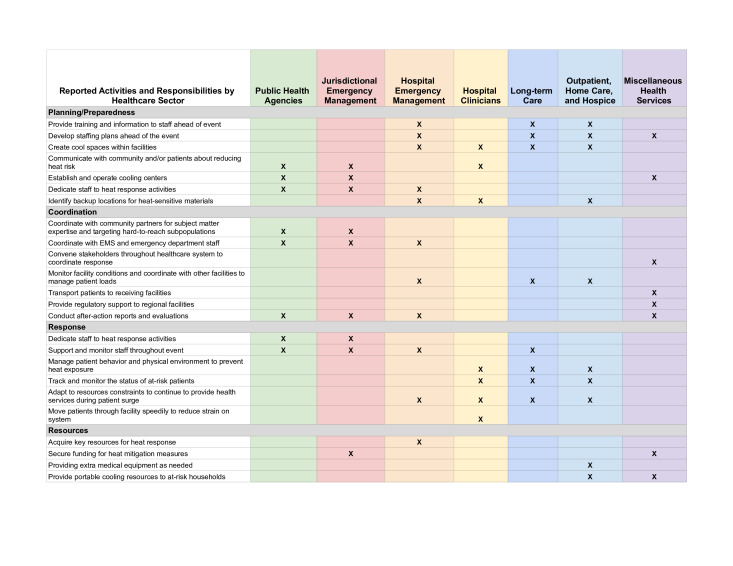
Extreme heat-related activities taken by and responsibilities of the various organisation types that make up the health sector, aligning with our allocation of organisation types to focus groups. Only responsibilities or activities explicitly reported in the focus group discussions are included. EMS, emergency medical systems.

While heat dome response varied depending on local conditions (eg, jurisdictional differences between emergency response agencies, rural vs urban setting, organisational resources and perceived level of public trust in the healthcare system), figure 1 presents reported heat response activities and responsibilities. Variations in local socio-economic, climatological and environmental conditions, combined with variety in the number of days with high temperatures, contributed to organisation-level impacts. Participants reported experiences ranging from “*we hadn’t seen a surge like this before*” to “*we had no impacts whatsoever in any of our facilities*”. Impacts on patients, staff and local communities stressed the health sector’s ability to provide services (table 1).

**Table 1:** Reported heat dome impacts by focus group

Focus group	Reported extreme heat event impacts
Public health agencies	Deaths among those living alone without air-conditioning Mass vaccination sites closed Moderate damage to equipment located on hot asphalt (eg, COVID-19 vaccination station)
Jurisdictional emergency management	Heating, ventilation, and air conditioning (HVAC) technicians overwhelmed by numerous HVAC failures due to lack of routine maintenance Unable to open COVID-19 vaccine/testing sites First responders fatigued
Hospital emergency management	Increase in heat-related illnesses Community members seeking shelter at the hospital Increased violence, aggression and disruptions attributed to the interaction of extreme heat and drug use Staff fatigued by heat and workload Heat-related electrical and power failures, medical equipment failure, temperature control system failures and water system issues Ambulance backup due to high patient load
Hospital clinicians	Infrastructure failure at laboratory delayed results turnaround Influx of critically ill patients due to heat-related illnesses Increased overnight capacity Difficulties treating the most at-risk patients (eg, older adults) due to patient surge and stretched resources
Long-term care facilities	Mood issues among patients Heat stress attributed to lack of ventilation Older and medically fragile patients exhibit lower heat tolerance
Home healthcare, hospice and outpatient services	Appointment cancellations Worsening health status attributed to heat impacts (eg, becoming bed-bound) Increased visits from nurses and home health aides to patient homes
Miscellaneous health services	Adapted cooling methods for patients in medical transports affect patient care provision Hot air and elevation affect the lift of aircraft/helicopters transporting patients Evacuation of patients from skilled nursing and long-term care Dehydration of patients and staff Staff equipment requirements (eg, gowns and flight suits) increase body temperature, contributing to fatigue Patients, community and staff suffer from heat-related mood dysregulation Lack of home resources compounds staff fatigue

### Heat dome impacts

Reported impacts on patients and community members included acute heat-related illnesses, complications of existing conditions (eg, heat exacerbating a patient’s chronic illness, rendering them bedbound) and behavioural changes. Participants indicated that populations with access and functional needs (eg, medically fragile, people over 65 years old, individuals who are isolated or unhoused, people with disabilities) experienced heightened risks of injury and death during the event. Per one long-term care worker, during EHEs “*our people die, and they die fast*.”

Heat also affected the variety and quality of healthcare service provision. Some participants reported significant heat exposure both at work, with high indoor temperatures and PPE that made cooling off difficult, and at home, where many lacked ways to cool down. This fatigued staff and strained their caregiving capabilities. Several local public health and emergency response agencies faced trade-offs between service provision and heat response, reassigning COVID-19-focused staff from testing and vaccination sites to heat response for additional capacity. The influx of heat-related patients also threatened to overwhelm organisational capacity and was problematic for organisations without staffed bed availability, with several participants echoing, “*It’s not the heat, it’s the surge*.”

The duration and intensity of the EHE taxed heat response resources, especially for healthcare providers; basic resources like ice were quickly used up and difficult to replace on short notice. Prolonged exposure to heat and increased usage also impacted infrastructure: participants reported failed temperature control systems, computer server room brownouts, water system issues and lab equipment failures. A common refrain was that a longer event would have overwhelmed capabilities.

Compounding and cascading hazards such as wildfires, wildfire smoke exposure and power outages were infrequently reported, though participants predicted more severe consequences in future events. According to one, *“In some ways, I think we were kind of lucky. I mean, we didn't see regional blackouts or brownouts. I think that was something I was really concerned about. In the future, if we do have that on top of extreme heat, kind of to this magnitude, I think it would be very different and very difficult for us to kind of cope.”* The event raised many participants’ awareness of the need to incorporate improved EHE planning into all-hazards planning.

### Barriers and facilitators to preparedness and response

**Figure 2:**
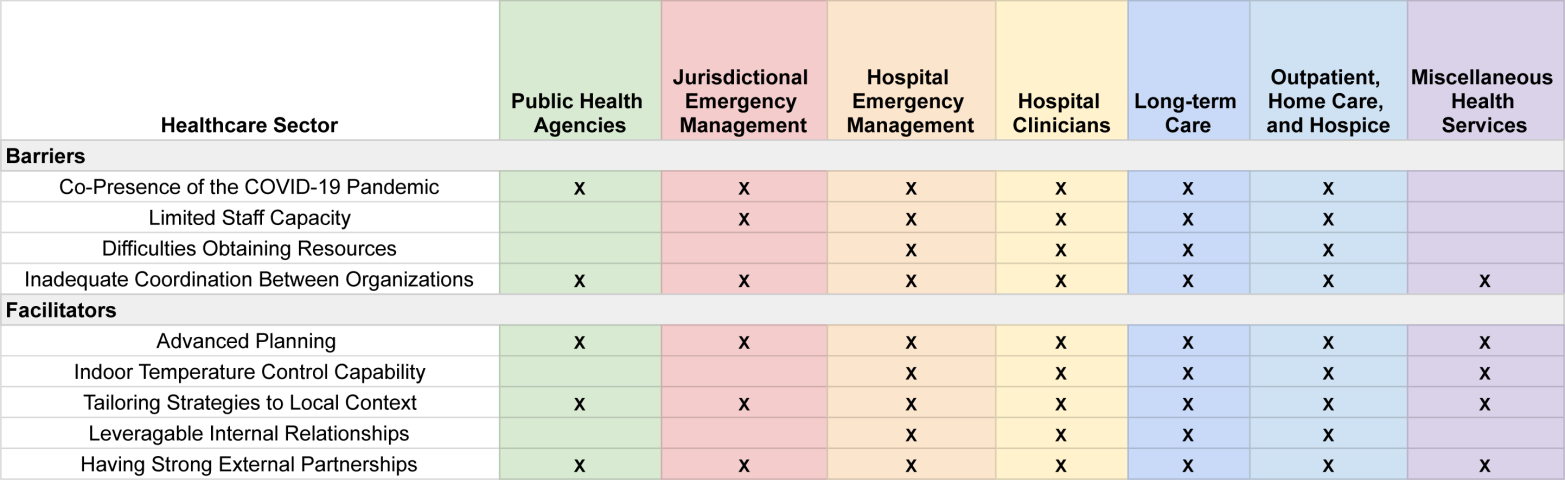
Barriers and facilitators of effective extreme heat event response reported by focus groups, as well as the focus groups that explicitly discussed these factors. An X is used to denote the presence of the barrier or facilitator.

Focus group participants identified barriers to preparedness and response, such as the co-presence of the COVID-19 pandemic, limited staff capacity, scarce resources and inadequate interorganisational collaboration; as well as facilitators, including advance planning, indoor temperature control, strategies tailored to patient and community preferences, internal relationships that could be leveraged to support staff and a strong network of external partners (figure 2).

#### Barriers

##### Co-presence of the COVID-19 pandemic

Focus group participants consistently acknowledged the COVID-19 pandemic as an implementation challenge. Although Washington State was lifting restrictions, several participants reported high local case rates; some organisations continued protective measures for patient-facing staff. Many facilities were already at or near capacity before the onset of the heat dome due to strain on the health system from the ongoing COVID-19 pandemic, which limited bed spaces and staff capacity during the heat-related surge. One participant observed, *“[We can’t] discharge to long term care and skilled nursing facilities because they don't have enough staff.”* Others described tensions between continuing COVID-19 protections, such as masking and removing fans from rooms, and heat protection.

##### Limited staff capacity

Staff burnout and shortages related to COVID-19 eroded baseline capacity, which participants reported was further degraded by a lack of available organisational expertise in heat response and heat-related healthcare needs, planned absences (eg, summer vacations) and volunteer shortages. This constrained response options and caused confusion about roles and responsibilities. Reduced capacity challenged staff’s ability to provide timely, specialised care for medically fragile individuals, particularly those requiring time-intensive care.

##### Difficulties obtaining resources

Supply chain issues, labour shortages, the novelty of the event and insufficient funding for heat response inhibited obtaining supplies and performing preventative infrastructure maintenance for some organisations. Several participants reported competing with the public for scarce heat response resources, such as fans and air conditioners, impacting their organisational readiness for heat response.

##### Inadequate interorganisational coordination

Inadequate coordination between organisations also challenged preparedness and response. Participants described an absence of coordinated regional communication with both peer and non-peer organisations, resulting in missed opportunities to compare and align response activities, share resources, control patient flow and engage hard-to-reach populations. Furthermore, several criticised ‘diversion’, a practice whereby facilities report to EMS being at capacity and refuse additional patients, thus ‘diverting’ patients to nearby facilities. Diversion was perceived to result in patients cycling through overburdened facilities unable to treat them.

### Facilitators

#### Advance planning

Participants expressed that advance planning allowed organisations to prepare as soon as the EHE was forecasted. Few reported already having heat-specific response plans, though some mentioned adapting emergency response and other hazard-related plans (eg, mass casualty event plans) or creating staffing and treatment plans immediately before the event. Several indicated that such plans improved their heat responses. Participants frequently referenced how tip sheets and other information shared by trusted expert sources (eg, NWHRN, local National Weather Service offices, and state and county agencies) guided their response planning.

Sharing information and resources with community members before the event was perceived to reduce pressure on the healthcare system. Emergency response agencies engaged with communities through app-based notifications, social media, websites, phone calls and emails. Some healthcare organisations used a tiered system to prioritise communications with at-risk populations (eg, home hospice patients). Others leveraged existing clinical encounters, including check-ups and telehealth visits, to share information and check on patients throughout the event.

Table 2 describes the resources identified as beneficial to heat response. Although scarce resources reportedly impeded response activities among some organisations, others acquired key resources ahead of the event due to advance planning.

**Table 2:** Important resources identified by focus group participants

Resources for extreme heat event response	Examples
Information	Just-in-time training EHE forecasting Temperature control strategies Tip sheets on extreme heat health impacts and response strategies Supply inventories Existing vulnerabilities and needs (facility and community) Experiences of prior extreme weather events by those in an organisation’s professional network
General supplies	Ice Water Popsicles Personal misters
Medical equipment and supplies	Ventilators Pharmaceuticals Intravenous drips
Cooling technology	HVAC Swamp coolers Fans Misters
Cooling centre amenities	Activities (eg, colouring books and games) Wi-Fi Snacks and water
Space	Cooled indoor spaces for patients Rest areas for staff Bed availability Temperature controlled storage
Funding	Grants from preparedness programmes COVID-19 funds

#### Indoor temperature control capabilities

Regulating indoor temperatures was reportedly crucial to providing healthcare services. Some organisations designated smaller areas to precool and maintain during the event. AC was identified by many as the premier cooling resource, though others used heat pumps, low-cost alternatives like box fans with ice in front, evaporative coolers, closed windows, and sun-blocking shades or blankets.

#### Tailoring strategies to local context

Implementing patient-centred strategies (‘recipient-centeredness’ in the Updated CFIR)^48^ helped many participants promote patient safety during the event. Hydration stations and cold treats (eg, popsicles), ice-lined body bags, misters, personal fans and spray bottles were described as low-cost solutions to managing patient temperatures. Many participants recounted high degrees of self-efficacy in implementing these measures and responding to the challenge of resource and operational constraints; one described “*MacGyvering*” solutions to patient needs with limited resources, and several shared the sentiment that “*we come together and take care of what needs to be taken care of.”*

Public health and emergency management organisations incentivised protective action through context-dependent strategies (‘local conditions’ in the Updated CFIR)45, including offering free movies in AC community buildings, free transit to or parking around cooling centres and basic resources such as water, snacks, Wi-Fi and entertainment (eg, colouring books and television) at cooling centres. Cooling centre utilisation varied; participants described how aligning location, services and familiarity with community needs and preferences increased utilisation. Several participants revised their cooling centre strategies post-heat dome, with some reconsidering locations and adopting alternative policies (such as loaning out AC units instead).

#### Addressing staff needs

Focusing on staff needs (‘deliverer-centeredness’ in the Updated CFIR)^48^ reportedly enhanced staff effectiveness by reducing fatigue and providing resources not otherwise available. Several organisations added or reassigned staff to increase capacity. Providing cool rest spaces for employees supported staff dealing with challenging conditions on shift and at home, leaving them more capable over time.

#### Strong external partnerships

Participants reported leveraging external collaborators (table 3) to educate and train both staff and communities on heat-related health impacts, establish cooling centres, conduct community outreach, share resources, acquire subject matter expertise and share information to establish situational awareness.

**Table 3:** External partnerships and reported benefits

External partners	Reported benefits	Regional examples
Local healthcare coalitions	Gather partners to facilitate conversations about extreme heat event preparedness and response Convene healthcare emergency managers to coordinate patient level loading	Northwest Healthcare Response Network
Local emergency response systems	Coordinate and allocate patient loads Interface between emergency response agencies and healthcare providers	Emergency medical services, emergency operation centres and disaster medical coordination centres
Private sector	Assist in resource acquisition Set up cooling shelters	HVAC businesses
Community-based organisations	Assist in resource acquisition Set up cooling shelters Provide volunteers to healthcare organisations (eg, ‘street paramedic programmes’) Tailor messaging for and engage with communities with access and functional needs (eg, people with limited English proficiency, people with disabilities and people living unhoused)	Faith-based organisations
External support organisations	Bolster organisational capacities	Health-based non-profits, transit organisations, mutual aid networks and transfer agreements
Local/state/federal governments and agencies	Provide information and resource support	National Weather Service, local emergency management agencies, local public health agencies
Utilities	Manage critical resources (power and water)	Local electrical utilities, local water utilities

Many relationships were built or strengthened because of regional collaboration during the COVID-19 pandemic. As one participant summarised, *“The relationships we've built with organizations and agencies through the COVID response have really, I think, strengthened our regional preparedness and our ability to respond.”*

## Discussion

Our findings reflect several common features of the heat dome response in western Washington’s health sector and the region’s ability to respond to future EHEs. The heat dome’s unprecedented nature was evident across all focus groups; participants established that the scale, intensity and duration of the event exposed systemic issues for the region, including anticipating and managing the patient surge, insufficient cooling capabilities in the built environment and the need for heat-specific planning. The *“building the plane while flying it”* approach may have compounded other issues, such as competing with the public for scarce cooling resources (eg, ice or AC support), difficulties managing bed availability and insufficient staff capacity. The few organisations with established heat action plans or all-hazards plans with relevant capabilities used the time from when the forecast was delivered (7–10 days) to prepare, indicating the importance of advance planning. However, findings suggest that the heat dome represented a paradigm shift for the region’s health sector, with increased awareness in some organisations and experience-informed preparedness actions taken by multiple others.

The influence of the COVID-19 pandemic and the threat of additional hazards on preparedness and response has implications for future polycrises. Although the pandemic placed physical and logistical strain on the health sector, most participants reported that pandemic collaborations had strengthened their networks and helped establish channels for resource and information sharing; responding to the pandemic’s uncertainties seemed to reinforce an attitude that the sector was now more capable of responding to unprecedented hazards. Additionally, while COVID-19 case rates were generally low in the region during the event, the trade-offs between COVID-19 and heat protection measures, the lack of bed spaces and other amplified pandemic-related stressors suggest that the heat dome’s health impacts could have been magnitudes higher had it co-occurred with a COVID-19 surge. The region was also fortunate not to have experienced compounding or cascading hazards, which remain a major concern for future EHEs. Given the importance of an adequate power supply for both cooling indoor spaces and continuing health services, concurrent blackouts and brownouts are of particular concern for the sector.^14 23 24^

Participants also described a tension: while a strong network of collaborators facilitated effective response, the lack of centralised coordination impeded it. Most focus groups identified the need for broader stakeholder convening and resource sharing. Jurisdictional variations in EHE responsibility distribution further challenged coordination. For example, emergency management agencies are responsible for coordinating cooling centres in some jurisdictions, while that responsibility falls to local health agencies in others. Bridging silos, sharing information to coordinate responses and increasing resource awareness will be important in future events.

### Contributions to literature

Our findings align with prior research on large-scale, long-duration EHEs, which suggests that healthcare services are in greater demand,^20^ service delivery is impacted when health workers face the same hot conditions as the communities they serve,^22^ managing COVID-19 can complicate heat response efforts and force trade-offs between COVID-19 and heat protection,^61 62^ and heat waves can occur when capacity is reduced due to staff leave.^63^ Furthermore, we confirm prior work that reducing the impacts of future EHEs will require individual behaviour change, as well as changes at the policy and system level, and throughout the built environment.^1564^,^66^

While participants did not explicitly acknowledge evidence informing their strategies outside of knowledge resources shared through trusted channels, many of the reported strategies align with evidence-based interventions; this suggests that there is at least regional-level awareness of effective approaches. Simultaneously, filling knowledge gaps related to optimal resource deployment, factors influencing partnerships and collaboration, relevant local context and the effectiveness of interventions in a timeframe useful to practitioners would improve EHE response. Improved processes for real-time collection and analysis of morbidity and mortality data are necessary to support associated research. Reflecting our finding that co-occurrence of other hazards is a major concern for future events, research into regional capacity to deal with supply chain disruptions, blackouts and brownouts, and compounding hazards is a priority.

### Recommendations for health sector preparedness and response

This research has important implications for improving EHE response in western Washington’s health sector. Recommendations for policymakers and practitioners are reported in online supplemental material 5. Centralised regional collaboration across preparedness, response and evaluation is possible and necessary. A state agency or non-profit is well positioned to host resources like a ‘one-stop heat shop’ for information about preparedness and response resources, best practices and forecasted events; a regional asset awareness or sharing platform; and discussions convening representatives from across the health sector before and after EHEs.

Organisations can produce co-benefits while improving preparedness for longer and compounding events. For instance, heat and cold planning share challenges like temperature control, and there are opportunities to make investments (eg, generators and improved temperature control systems) that improve resilience to both. Investments in the built environment, both in facilities and at the community scale, are increasingly necessary to adapt to the region’s changing climate. Organisations can also adapt existing practices for heat response, such as developing patient heat-risk assessment intake forms and using patient check-ins or telehealth to share information and resources about upcoming EHEs. Establishing community contact early and often can reduce patient surge and related stress on the health sector. Turnover and reduced staff capacity have changed the health sector since the COVID-19 pandemic; incorporating redundancies and clearly delineating response roles that anyone can ‘step into’ during staff shortages is one possible stopgap.

Improving and systematising evaluative processes will help implement these recommendations. Interorganisational relationships must be formalised and made resilient to changes in staff or leadership. Although the frequency of hazards in recent years has provided experiential knowledge, high rates of turnover and burnout reported by participants suggest that this knowledge needs to be institutionalised. Additionally, developing capacity for near-real-time excess morbidity and mortality data will improve the health sector’s ability to adjust response strategies during an event and before subsequent events. Finally, while participants were largely positive about the responsiveness of regional public health and emergency management organisations, evaluation of preparedness and response in the broader context of climate-sensitive hazards is necessary to adjust to changing environmental conditions.

### Limitations

Participants were part of NWHRN’s member organisations; considering our opt-in recruitment, some critical perspectives may be inadequately represented in our participant pool. These include facilities workers, smaller private practices, EMS providers, tribal health and community health workers. Our research focused exclusively on those within the health sector; the public’s perspectives were not included, potentially missing some gaps or challenges not observed by participants. Social desirability bias is possible; many participants have ongoing professional relationships and may have been hesitant to disclose organisational or interorganisational-level challenges. Additionally, focus groups occurred a year after the heat dome; while participants referred to contemporaneous notes or documents, recall bias remains a concern. NWHRN was represented in the focus groups through this paper’s authors; this association may have caused participants to modify their responses. Finally, care should be taken when applying findings to contexts outside of western Washington as robust regional response infrastructure may occlude challenges that would appear in other regions.

## Conclusion

The 2021 heat dome was unprecedented for western Washington, causing a shift in the EHE response paradigm. Despite significant challenges—many stemming from COVID-19 impacts, unfamiliarity with EHEs and struggles with coordination beyond existing silos—the health sector responded using scarce resources and innovated to continue providing essential health services. Reducing human and institutional impacts of future events will require significant effort to improve the region’s policies, plans and built environment across the health sector. Motivation, knowledge and awareness gained from the response remain high given the heat dome’s recency; rapidly institutionalising its lessons is critical. Investing in extreme heat resilience must be a high short-term and long-term priority as the frequency of such large-scale events is increasing.

## Supplementary material

10.1136/bmjopen-2024-089093 online supplemental material 1

10.1136/bmjopen-2024-089093 online supplemental material 2

10.1136/bmjopen-2024-089093 online supplemental material 3

10.1136/bmjopen-2024-089093 online supplemental material 4

10.1136/bmjopen-2024-089093 online supplemental material 5

## Data Availability

Data are available in a public, open access repository.
