# Supplementary material for: Lessons learnt from the 2021 Pacific Northwest heat dome: a qualitative study of western Washington’s healthcare community response
Source: BMJ Open. 2025 Apr 17;15(4):e089093. doi: 10.1136/bmjopen-2024-089093 (PMC12007061; doi:10.1136/bmjopen-2024-089093)
Supplement: online supplemental material 1 [file bmjopen-15-4-s001.pdf]

Supplemental Materials 1: Use of the Consolidated Framework for Implementation Research

The Consolidated Framework for Implementation Research (CFIR), which has been noted for its evaluative strength at identifying barriers and facilitators to implementation [1–3], was applied to assess the determinants of implementation of the suite of heat response strategies among healthcare organizations in western Washington state. The CFIR has a robust history of applications in a variety of contexts (at the time of research, it had been used in at least 376 published articles), though its primary use has historically been on clinical interventions [4]. At the time of this project's design phase, the vast majority of projects (>83%) that had used the CFIR were related specifically to healthcare, though the CFIR had also been applied in public health, education, and other contexts [4]. The authors of CFIR themselves note that many implementations evaluated using CFIR involve innovations that may be unfamiliar to the setting they are applied in, which certainly applies to a novel event for a region's health sector [5]. The CFIR had also recently been reorganized into its second iteration (the Updated CFIR), which gave us the opportunity to test its intended new flexibility in a context (a suite of extreme event response actions) that it had not previously been applied to [4]. This research thus also offers a contribution to the body of implementation science research by expanding the use cases of the CFIR beyond discrete interventions in healthcare.

Following the CFIR guidance, researchers MK and CH reviewed the 39 Updated CFIR constructs across its five domains: innovation (what is being implemented), outer setting (the broader context surrounding the implementation setting), inner setting (the implementation setting itself), individuals (roles and characteristics of individuals involved in innovation implementation), and implementation process (implementation strategies and activities) [6,7]. MK and CH used consensus decision-making to identify 22 constructs based on their likely influence on the implementation of EHE response activities using prior knowledge of the region's health sector and consultation with the other authors. While the focus group questions were largely open-ended and aligned broadly with one of the five CFIR domains, individual constructs were used primarily in data analysis as the foundation of the codebook and analytic matrix. Constructs from the innovation domain were ultimately excluded because they were a poor fit for a suite of related activities that cannot be easily separated. The roles subdomain of individual characteristics domain was similarly difficult to parse in analysis, as the participant pool of emergency management contacts were not easily separable into the provided constructs. Finally, given the fact that those implementing EHE response were themselves affected by both the extreme heat and the ongoing COVID-19 pandemic, MK and CH added additional context to the "critical incidents" constructs. A summary of the determinations made for each construct is included below, with definitions derived from the official 2022 Updated CFIR definitions [4]:

		Inclusion?	Justification	Notes
**I. INNOVATION DOMAIN**<br><br>**Project Innovation: The suite of available extreme heat event response activities available to the health sector.**		No.	MK and CH determined that this domain was a poor fit for a suite of interventions, as constructs largely evaluated singular interventions.	
**Construct Name**	**Definition**			
A. Innovation Source	The group that developed and/or visibly sponsored use of the innovation is reputable, credible, and/or trustable.			
B. Innovation Evidence-Base	The innovation has robust evidence supporting its effectiveness.			
C. Innovation Relative Advantage	The innovation is better than other available innovations or current practice.			
D. Innovation Adaptability	The innovation can be modified, tailored, or refined to fit local context or needs.			
E. Innovation Trialability	The innovation can be tested or piloted on a small scale and undone.			
F. Innovation Complexity	The innovation is complicated, which may be reflected by its scope and/or the nature and number of connections and steps.			
G. Innovation Design	The innovation is well designed and packaged, including how it is assembled, bundled, and presented.			
H. Innovation Cost	The innovation purchase and operating costs are affordable.			
**II. OUTER SETTING DOMAIN**<br><br>**Project Outer Setting(s): The broad region of western Washington, with a particular focus on the policies and institutions surrounding the region's health sector.**		Yes.	MK and CH determined that this domain would be useful to evaluate the context around and identify system-level/region-level barriers, facilitators, and lessons learned.	
**Construct Name**	**Definition**			
A. Critical Incidents	Large-scale and/or unanticipated events disrupt implementation and/or delivery of the innovation.	Yes.	Included because background research and listening sessions suggested that a key facet of heat response implementation is that it does not happen in a vacuum and that there is the potential for compounding disasters to occur	Added three pieces of local context when coding: challenges specific to the heat wave, the actual effects of the heat wave on participants and those they served, and the context of the ongoing COVID-19 pandemic
B. Local Attitudes	Sociocultural values (e.g., shared responsibility in helping recipients) and beliefs (e.g., convictions about the worthiness of recipients) encourage the Outer Setting to support implementation and/or delivery of the innovation.	No.	Excluded because listening sessions suggested that attitudes and beliefs at the regional level among participants were largely shared during a crisis.	Potential target for future research; understanding how prior preparedness activities and existing infrastructure has affected local atittudes related to preparedness and response activities
C. Local Conditions	Economic, environmental, political, and/or technological conditions enable the Outer Setting to support implementation and/or delivery of the innovation.	Yes.	Included because listening sessions suggested significant local variation in impacts and conditions.	
D. Partnerships & Connections	The Inner Setting is networked with external entities, including referral networks, academic affiliations, and professional organization networks.	Yes.	Included because listening sessions revealed the abundance of local partnerships (particularly through the lens of NWHRN collaboration), so this construct was noted as a key consideration	High co-occurence with resource-related constructs, particularly knowledge and information
E. Policies & Laws	Legislation, regulations, professional group guidelines and recommendations, or accreditation standards support implementation and/or delivery of the innovation.	Yes.	Included because the novel nature of the event offered the opportunity to investigate any barriers or facilitators related to existing policies and laws at the time of the heat dome	
F. Financing	Funding from external entities (e.g., grants, reimbursement) is available to implement and/or deliver the innovation.	No.	Excluded because researchers noted that participants were more involved with the inner setting equivalent (Available Resources/Funding)	
G. External Pressure	External pressures drive implementation and/or delivery of the innovation.<br>Use this construct to capture themes related to External Pressures that are not included in the subconstructs below.	No.	Excluded because researchers determined that the pressure to implement the innovations stemmed primarily from the need to respond to the emergency event	
1. Societal Pressure	Mass media campaigns, advocacy groups, or social movements or protests drive implementation and/or delivery of the innovation.			
2. Market Pressure	Competing with and/or imitating peer entities drives implementation and/or delivery of the innovation.			
3. Performance-Measurement Pressure	Quality or benchmarking metrics or established service goals drive implementation and/or delivery of the innovation.			
**III. INNER SETTING DOMAIN**<br><br>**Project Inner Setting(s): The individual organizations within the western Washington health sector.**		Yes.	MK and CH determined that this domain would be useful to evaluate the context around and identify organizational-level barriers, facilitators, and lessons learned.	
**Construct Name**	**Definition**			
Note:	*Constructs A – D exist in the Inner Setting regardless of implementation and/or delivery of the innovation, i.e., they are persistent general characteristics of the Inner Setting.*			
A. Structural Characteristics	Infrastructure components support functional performance of the Inner Setting.<br>Use this construct to capture themes related to Structural Characteristics that are not included in the subconstructs below.	Yes.	Included to identify barriers and facilitators related to the social and built environment that might not be captured elsewhere	
1. Physical Infrastructure	Layout and configuration of space and other tangible material features support functional performance of the Inner Setting.	Yes.	Included because listening sessions highlighted the importance of equipment and other existing infrastructure in temperature control and health service provision	
2. Information Technology Infrastructure	Technological systems for tele-communication, electronic documentation, and data storage, management, reporting, and analysis support functional performance of the Inner Setting.	No.	Excluded because information technology was not significantly discussed during the listening sessions; relevant power and equipment concerns are captured under physical infrastructure.	
3. Work Infrastructure	Organization of tasks and responsibilities within and between individuals and teams, and general staffing levels, support functional performance of the Inner Setting.	Yes.	Included because focus group participants were in a good position to describe division of labor when it came to implementing EHE response activities	

B. Relational Connections	There are high quality formal and informal relationships, networks, and teams within and across Inner Setting boundaries (e.g., structural, professional).	Yes.	Included to examine relations within an organization and other facilities within the same organization	
C. Communications	There are high quality formal and informal information sharing practices within and across Inner Setting boundaries (e.g., structural, professional).	No.	Excluded to streamline codebook; for the purposes of the research question, this content would likely be included in relational connections and external partnerships.	
D. Culture	There are shared values, beliefs, and norms across the Inner Setting.<br>Use this construct to capture themes related to Culture that are not included in the subconstructs below.	Yes.	Included primarily to capture the deliverer and recipient centeredness; these constructs would assess the how both health sector workers and users were viewed during the event	
1. Human Equality-Centeredness	There are shared values, beliefs, and norms about the inherent equal worth and value of all human beings.	No.	Excluded because listening sessions suggested that this was less relevant to decisionmaking among the the participant pool	
2. Recipient-Centeredness	There are shared values, beliefs, and norms around caring, supporting, and addressing the needs and welfare of recipients.	Yes.	Included to assess how service users/patients/community members were viewed in the context of EHE response activities	
3. Deliverer-Centeredness	There are shared values, beliefs, and norms around caring, supporting, and addressing the needs and welfare of deliverers.	Yes.	Included to assess how those implementing EHE response were viewed in the context of the whole-system implementation	
4. Learning-Centeredness	There are shared values, beliefs, and norms around psychological safety, continual improvement, and using data to inform practice.	No.	Excluded to streamline codebook because listening sessions indicated this construct was less relevant to the participant pool; MK and CH also determined that relevant information would be captured in the process domain (Reflecting and Evaluating).	
Note:	*Constructs E – K are specific to the implementation and/or delivery of the innovation.*			
E. Tension for Change	The current situation is intolerable and needs to change.	No.	Excluded because MK and CH determined that tension was less relevant in the context of a novel event	
F. Compatibility	The innovation fits with workflows, systems, and processes.	No.	Excluded to streamline codebook; MK and CH determined that this information was captured across other domains and that given the suite of interventions, the construct loses some of evaluative power (as organizations would be selecting the best-fit set of interventions).	
G. Relative Priority	Implementing and delivering the innovation is important compared to other initiatives.	Yes.	Included to assess balance of EHE response against other priorities (continuity of care, COVID-19 precautions, etc.)	
H. Incentive Systems	Tangible and/or intangible incentives and rewards and/or disincentives and punishments support implementation and delivery of the innovation.	No.	Excluded because incentives were not identified during listening sessions and because the construct was not a good fit based on researcher knowledge of local systems	
I. Mission Alignment	Implementing and delivering the innovation is in line with the overarching commitment, purpose, or goals in the Inner Setting.	No.	Excluded in light of MK and CH determination that reponse activities were necessary to carry out any organizational mission, thus losing some of the construct's explanatory power	
J. Available Resources	Resources are available to implement and deliver the innovation.<br>Use this construct to capture themes related to Available Resources that are not included in the subconstructs below.	Yes.	Included as listening sessions suggested this was a key component of response activities and cross-cutting concern	One of the most-applied codes when discussing response activities
1. Funding	Funding is available to implement and deliver the innovation.	Yes.	Included to identify funding sources and structures (or lack thereof) available to organizations as part of resource-related barriers and facilitators	
2. Space	Physical space is available to implement and deliver the innovation.	Yes.	Included to recognize potential opportunities or constraints related to resources in the built environment	Applied applied with respect to bed spaces and existing cool spaces
3. Materials & Equipment	Supplies are available to implement and deliver the innovation.	Yes.	Included to identify and understand what supplies were necessary to carry out EHE activities	
K. Access to Knowledge & Information	Guidance and/or training is accessible to implement and deliver the innovation.	Yes.	Included in order to understand what knowledge and information resources existed and were accessed by those responding to the event despite the event's novelty in the region	This also included information that was available to the public where participants noted it
**IV. INDIVIDUALS DOMAIN**<br><br>**Project Individuals: The roles and characteristics of individuals employed by organizations within the region's health sector. Given the diversity of participating organizations (and inner settings) and the organization-type arrangement of focus groups, this study focused primarily on the shared characteristics of individuals.**		Yes.	MK and CH determined that this domain would be useful to evaluate the context around and identify individual-level barriers, facilitators, and lessons learned.	
**ROLES SUBDOMAIN**		No.	Excluded as MK and CH determined this construct was largely redundant due to drawing on emergency management contacts for participant pool.	
**Construct Name**	**Definition**			
A. High-level Leaders	Individuals with a high level of authority, including key decision-makers, executive leaders, or directors.	[redacted]		
B. Mid-level Leaders	Individuals with a moderate level of authority, including leaders supervised by a high-level leader and who supervise others.			
C. Opinion Leaders	Individuals with informal influence on the attitudes and behaviors of others.			
D. Implementation Facilitators	Individuals with subject matter expertise who assist, coach, or support implementation.			
E. Implementation Leads	Individuals who lead efforts to implement the innovation.			
F. Implementation Team Members	Individuals who collaborate with and support the Implementation Leads to implement the innovation, ideally including Innovation Deliverers and Recipients.			
G. Other Implementation Support	Individuals who support the Implementation Leads and/or Implementation Team Members to implement the innovation.			
H. Innovation Deliverers	Individuals who are directly or indirectly delivering the innovation.			
I. Innovation Recipients	Individuals who are directly or indirectly receiving the innovation.			
**CHARACTERISTICS SUBDOMAIN**<br><br>**Project Characteristics: This study followed CFIR guidance by focusing primarily on the COM-B characteristics of individuals across roles and organizations.**		Yes.	Included given research need to understand individual decisionmaking and experiences within broader organizational contexts	
**Construct Name**	**Definition**			

A. Need	The individual(s) has deficits related to survival, well-being, or personal fulfillment, which will be addressed by implementation and/or delivery of the innovation.	No.	Excluded as MK and CH determined that deficit-based need was not a likely barrier/facilitator given the emergency context, selection of emergency management contacts as participants and opt-in nature of the participants	
B. Capability	The individual(s) has interpersonal competence, knowledge, and skills to fulfill Role.	Yes.	Included because of the event's novel nature of event; MK and CH determined that this construct was likely to offer insights into barriers and facilitators related to implementation	
C. Opportunity	The individual(s) has availability, scope, and power to fulfill Role.	Yes.	Included because of the competing priorities and resource limitations during the event; MK and CH determined that this construct was likely to offer insights into barriers and facilitators related to implementation	
D. Motivation	The individual(s) is committed to fulfilling Role.	No.	Excluded as MK and CH determined that motivation was not a likely barrier/facilitator given the emergency context, selection of emergency management contacts as participants and opt-in nature of the participants	
**V. IMPLEMENTATION PROCESS DOMAIN**<br><br>**Project Implementation Process: The advanced planning, procedures, and other activities and strategies used to deliver a suite of extreme heat event response activities.**		Yes.	MK and CH determined that this domain was a key component of the evaluation when looking across a suite of interventions, and could serve as a key point of comparison between organizations.	
**Construct Name**	**Definition**			
A. Teaming	Join together, intentionally coordinating and collaborating on interdependent tasks, to implement the innovation.	No.	Excluded because participants tended to speak about their individual activities; MK and CH determined that his was somewhat masked when viewing a suite of interventions	
B. Assessing Needs	Collect information about priorities, preferences, and needs of people.<br>Use this construct to capture themes related to Assessing Needs that are not included in the subconstructs below.	No.	Excluded to streamline codebook; MK and CH determined that the information collection stage was largely coverd by other constructs where relevant (e.g. Engaging or Planning)	Would have a larger presence with a greater focus on patients/health service users.
1. Innovation Deliverers	Collect information about the priorities, preferences, and needs of deliverers to guide implementation and delivery of the innovation.			
2. Innovation Recipients	Collect information about the priorities, preferences, and needs of recipients to guide implementation and delivery of the innovation.			
C. Assessing Context	Collect information to identify and appraise barriers and facilitators to implementation and delivery of the innovation.	No.	Excluded to streamline codebook; MK and CH determined that the information collection stage was largely coverd by other constructs where relevant (e.g. Engaging or Planning)	Would have a larger presence with a greater focus on patients/health service users
D. Planning	Identify roles and responsibilities, outline specific steps and milestones, and define goals and measures for implementation success in advance.	Yes.	Included due to MK and CH determination that advanced planning and organizational roles were a key topic of consideration, particularly given the novel nature of the event	
E. Tailoring Strategies	Choose and operationalize implementation strategies to address barriers, leverage facilitators, and fit context.	Yes.	Included to assess how participants adjusted suite of interventions for their local context	
F. Engaging	Attract and encourage participation in implementation and/or the innovation.<br>Use this construct to capture themes related to Engaging that are not included in the subconstructs below.	Yes.	Included only the high-evel construct for codebook brevity; this construct covered information-seeking activities as well as participation (both deliverers and recipients) in implementation of heat response ativities	
1. Innovation Deliverers	Attract and encourage deliverers to serve on the implementation team and/or to deliver the innovation.			
2. Innovation Recipients	Attract and encourage recipients to serve on the implementation team and/or participate in the innovation.			
G. Doing	Implement in small steps, tests, or cycles of change to trial and cumulatively optimize delivery of the innovation.	No.	Excluded as the construct was not as relevant when considering the novel nature of the heat dome and the evaluation of the implementation of a suite of interventions	More relevant for an investigation of EHE response across several heat seasons
H. Reflecting & Evaluating	Collect and discuss quantitative and qualitative information about the success of implementation.<br>Use this construct to capture themes related to Reflecting & Evaluating that are not included in the subconstructs below.	Yes.	Included given interest in lessons learned; discussions around after-action reporting/existing evaluation processes were a high priority.	
1. Implementation	Collect and discuss quantitative and qualitive information about the success of implementation.			
2. Innovation	Collect and discuss quantitative and qualitative information about the success of the innovation.			
I. Adapting	Modify the innovation and/or the Inner Setting for optimal fit and integration into work processes.	No.	Excluded to streamline codebook; MK and CH determined that the relevant information would be captured by the Tailoring Strategies construct.	

References:

1 Safaeinili N, Brown-Johnson C, Shaw JG, *et al.* CFIR simplified: Pragmatic application of and adaptations to the Consolidated Framework for Implementation Research (CFIR) for evaluation of a patient-centered care transformation within a learning health system. *Learn Health Syst*. 2020;4:e10201.

2 Lam H, Quinn M, Cipriano-Steffens T, *et al.* Identifying actionable strategies: using Consolidated Framework for Implementation Research (CFIR)-informed interviews to evaluate the implementation of a multilevel intervention to improve colorectal cancer screening. *Implement Sci Commun*. 2021;2:57.

3 Kirk MA, Kelley C, Yankey N, *et al.* A systematic review of the use of the Consolidated Framework for Implementation Research. *Implement Sci*. 2016;11:72.

4 Damschroder LJ, Reardon CM, Widerquist MAO, *et al.* The updated Consolidated Framework for Implementation Research based on user feedback. *Implement Sci*. 2022;17:75.

5 Damschroder LJ, Aron DC, Keith RE, *et al.* Fostering implementation of health services research findings into practice: a consolidated framework for advancing implementation science. *Implement Sci*. 2009;4:50.

6 Updated CFIR Constructs. https://cfirguide.org/constructs/ (accessed 14 November 2024)

7 Overview. https://cfirguide.org/evaluation-design/overview/ (accessed 14 November 2024)
