# Supplementary material for: Lessons learnt from the 2021 Pacific Northwest heat dome: a qualitative study of western Washington’s healthcare community response
Source: BMJ Open. 2025 Apr 17;15(4):e089093. doi: 10.1136/bmjopen-2024-089093 (PMC12007061; doi:10.1136/bmjopen-2024-089093)
Supplement: online supplemental material 2 [file bmjopen-15-4-s002.docx]

**Supplemental Materials 2: NWHRN Listening Session and Focus Group Participant Pool**

NWHRN hosts monthly listening sessions for its member organizations on a variety of topics of interest. At the time of this study, NWHRN’s service area included health sector organizations from 15 counties in western Washington, as well as 24 tribal nations in the region. The map below provides an overview of this coverage for additional context.

**NHWRN Service Area (Counties and Tribal Nations)**


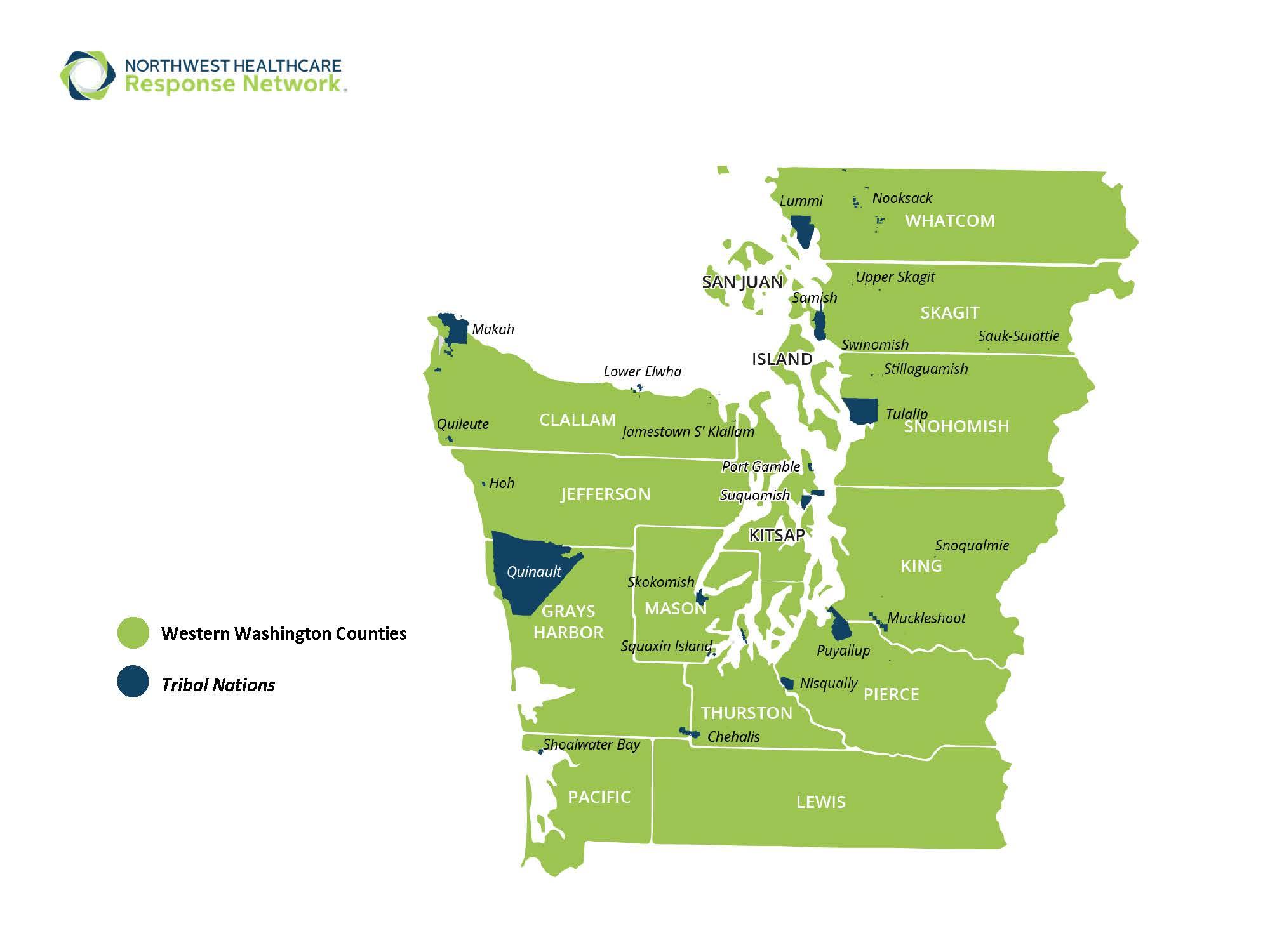


***Counties:*** *Clallam, Grays Harbor, Island, Jefferson, King, Kitsap, Lewis, Mason, Pacific, Pierce, San Juan, Skagit, Snohomish, Thurston, and Whatcom*

***Tribal Nations:*** *Chehalis,* *Hoh, Jamestown S’Klallam, Lummi, Makah, Muckleshoot, Nisqually, Nooksack, Quileute, Quinault, Port Gamble, Puyallup, Samish, Sauk-Suiattle, Shoalwater Bay, Skokomish, Snoqualmie, Squaxin Island, Stillaguamish, Suquamish, Swinomish, Tulalip, Upper Skagit*

In order to adequately address the diverse needs of such a large community of practice, NHWRN divides the counties up into four service districts: North, Northwest, West, and Central. These service districts are depicted below:

**NWHRN Service Districts (2022)**


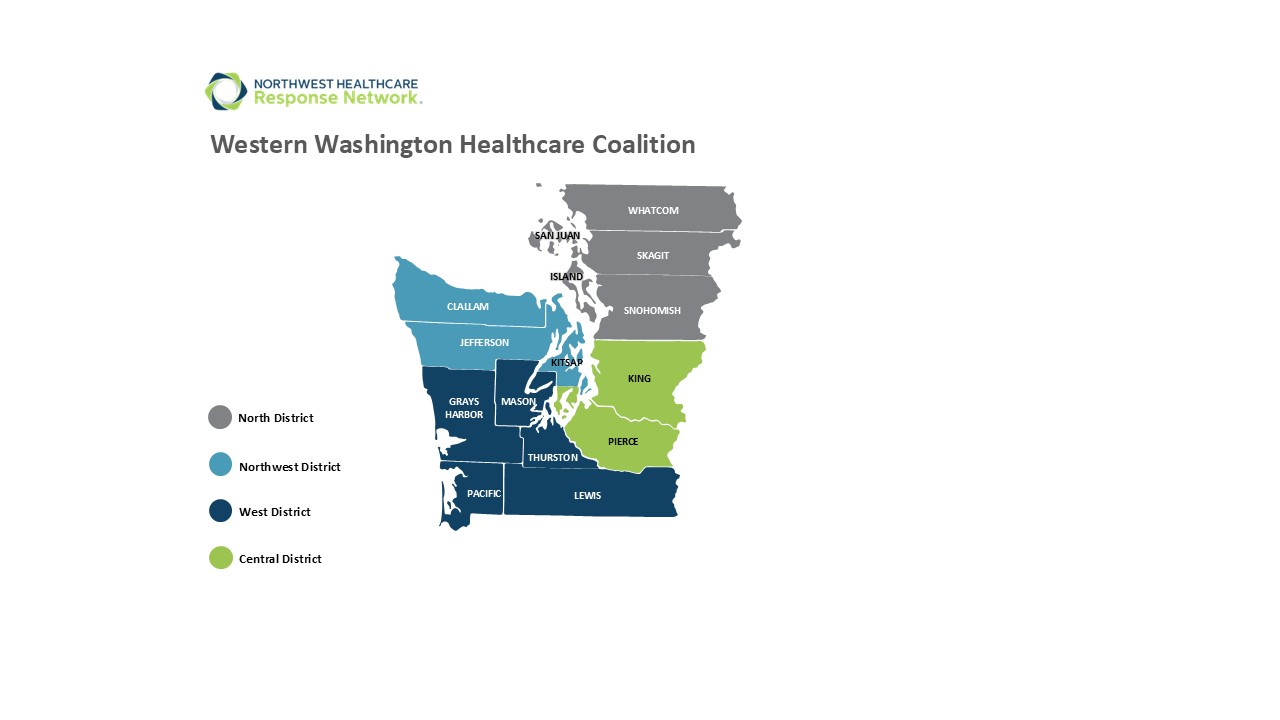


***North District:*** *Island, San Juan, Skagit, Snohomish, and Whatcom*

***Northwest District:*** *Clallam, Jefferson, Kitsap*

***West District:*** *Grays Harbor, Lewis, Mason, Pacific, Thurston*

***Central District:*** *King, Pierce*

NWHRN’s monthly listening sessions are attended by those with a specific interest in the monthly topic; as such, the audience shifts between listening sessions. While typical participants include a wide range of organization roles, from emergency managers, executive leadership and physicians depending on the topic. For example, had the research questions and NWHRN’s interest been on patient impacts during the EHE, NWHRN would have reached out to other points of contact at the member organizations and consulted with their clinical advisor to support the outreach. However, the listening sessions that informed this project were specifically tailored towards individuals who were likely to have participated in a suite of heat response activities.

Given the nature of the response and the dual purpose of the listening sessions (informing both this research and NWHRN’s after-action reporting), NWHRN invited the emergency management contacts they had at each of their member organizations to attend, as they would have been NWHRN’s point of contact during the heat dome. Member organizations that participated included local health jurisdictions, county emergency management, tribal partners, hospitals, long-term care facilities, outpatient clinics, surgery centers, fire and emergency medical services, homecare and hospice providers, regulatory bodies, and several health-related nonprofits that played a response role. Some organizations, like smaller family medicine physicians, may not have been represented on the call due to both the research focus and the need for an emergency management contact shared with NWHRN.

The points of contact who attended the sessions were generally dedicated emergency managers (or equivalent) for their organizations, with some nursing, security, and/or safety staff included when they functioned as the emergency managers for their facilities. Participants thus shared perspectives from a variety of healthcare roles, including health administration, primary medical care and healthcare provision, public health, and emergency management.

After the listening sessions were held, the research team worked to identify members who would have the capacity and perspectives to participate in the follow-on focus groups that would serve as the data for this study. Using the same emergency management contact list and accounting for attendance and participation at the listening session, the type of health organization, and the NWHRN service area; the research team compiled a list of potential participants that covered the breadth of organization types involved in the listening sessions and were balanced across service areas. The team invited these contacts to participate using a formalized email template. Participants who opted to participate were then grouped by organizational type; some of the less common organization types (for example, home health care and outpatient centers) were combined with others with similar responsibilities to ensure quorum at the focus groups. While focus groups were initially scheduled with between six and eight anticipated participants, day-of dropouts reduced the number of participants in each group to between three and six. Missing participant perspectives as a result of the opt-in structure and day-of dropouts are discussed as some of the limitations of this research.
