# Supplementary material for: Lessons learnt from the 2021 Pacific Northwest heat dome: a qualitative study of western Washington’s healthcare community response
Source: BMJ Open. 2025 Apr 17;15(4):e089093. doi: 10.1136/bmjopen-2024-089093 (PMC12007061; doi:10.1136/bmjopen-2024-089093)
Supplement: online supplemental material 3 [file bmjopen-15-4-s003.docx]

**Supplemental Materials 3: Focus Groups Facilitator Guide**

# Welcome

- Thank you for agreeing to participate in our focus group today. My name is [Facilitator Name] and I am a [role] in the [organization]. I am part of a Northwest Healthcare Response Network (NWHRN) and University of Washington team working to understand how the healthcare community in the Pacific Northwest experienced the June 2021 heat wave, and the heat wave this summer.
- The purpose of this discussion is to help us understand the ways in which the healthcare community was stressed by the June 2021 heat wave, strengths of the response and areas for improvement. While we recognize that there have been other heat waves in the region since June 2021, we will be focusing on that event specifically because temperatures were so extreme and lasted for several days. For context, in the last week of June of 2021 the Pacific Northwest region of the US and parts of B.C. Canada experienced an extreme heat event with unusually high temperatures lingering for several days. Temperatures exceeding 104 ºF occurred between Sunday June 27 to Tuesday June 29,2021, with temperatures topping 110ºF in parts of Washington. At least 100 heat-related deaths occurred in the state, and there was a spike in ED visits across the region during this time. The June 2021 heatwave was considered a one in a 1,000 year event, but these types of extreme events are expected to become more common as the planet warms.

# Explanation of the process

- We are using a focus group format to encourage an in-depth discussion among you all. We are seeking to learn from you the ways in which the healthcare community was stressed by the June 2021 heat wave and heat events this past summer, and to identify strengths of the response and areas for improvement. There are no wrong answers. We’re not trying to achieve consensus; we’re gathering information.
- The session will last one hour.
- We will be taking notes and recording so that we can refer to the discussion later.
- We may write up our findings in a report or for publication in a peer-reviewed journal. We will not refer to you by name or agency in any report or publication without your prior explicit permission.
- Your participation is voluntary. You can refuse to answer any question, and you can leave the session at any time. You will not be penalized for not answering any question or for leaving the session.

# Ground Rules

- We do have a few ground rules. We hope everyone will participate and chime-in during the discussion. Information provided in the discussion must be kept confidential. Please do not share what was said or who was here. Please stay with the group and avoid distractions. We will also be utilizing the chat feature today, so please feel free to add any thoughts in the chat box throughout the discussion.

#

# Questions and consent

- Does anyone have any questions before we begin?
- Do you consent to participate in this focus group and to this discussion being recorded? [Ask everyone to provide a verbal “yes”]
- Turn on Tape Recorder
- One more time, do you consent to participate in this focus group and to this discussion being recorded? [Ask everyone to provide a verbal “yes”]

Before we begin, can you please share your first name, your role, and your organization in the chat?

**BACKGROUND/EVENT IMPACTS**

- **[OUTER SETTING]** Can you describe the impacts experienced by your organization and those it served during the 2021 heat wave and heat events this past summer?

**ORGANIZATIONAL RESPONSE**

- **[INNER SETTING]** What were the primary challenges you faced while responding to the heat wave of 2021 and subsequent heatwaves?
  - *Prompts: resource needs, capacity (esp. staff), vulnerable populations*
- **[INNER SETTING]** What did your organization do well during the June 2021 heat wave response?
  - **[READINESS FOR IMPLEMENTATION]** Follow Up: What helped prepare you for the event?
    - *Prompts: incident command, contact sheets, early warning and alert systems, tip sheets, partnerships*
- **[OUTER SETTING]** What relationships outside of your organization were most helpful to your response?
  - *Prompts: NWS, relationships built through COVID response*
- **[OUTER SETTING]** How aware are members of the staff at your organization of the needs and preferences of the community members served, and how well do you think your organization met those individual’s needs during the 2021 and subsequent heatwaves?
  - Follow Up: Describe any barriers that individuals experienced being served by your organization during the 2021 heatwave and subsequent heat events.

**EVALUATION AND LESSONS LEARNED**

- **[PROCESS]** During and after the 2021 heat wave, what evaluation methods did your organization use to assess response efforts?
  - *Prompt: After Action Reports, informal internal discussions,*
  - **Follow Up:** Did you start to do these leading into this past summer?
- **[PROCESS: REFLECTING AND EVALUATION]** What are the key lessons learned from the 2021 heat wave and how has your organization adjusted your preparedness planning for future events?
  - *Prompt: formal change of plans, applying for grant, strengthening/developing networks*
  - **Follow Up: [CHARACTERISTICS OF INDIVIDUALS]** After last summer’s heat wave response, how confident do you feel that your organization is ready for future heat events?
- **[READINESS FOR IMPLEMENTATION]** What resources, tools, networks, and other support would be helpful for your response to future heat waves?
  - *Prompt: funding for AC units/box fans, updated contact lists, etc.*
