# Supplementary material for: Lessons learnt from the 2021 Pacific Northwest heat dome: a qualitative study of western Washington’s healthcare community response
Source: BMJ Open. 2025 Apr 17;15(4):e089093. doi: 10.1136/bmjopen-2024-089093 (PMC12007061; doi:10.1136/bmjopen-2024-089093)
Supplement: online supplemental material 4 [file bmjopen-15-4-s004.pdf]

Supplemental Materials 4: Codebook

Code Name	Parent code (if applicable)	NVIVO Shorthand	Related domain	Definition	Inclusion/ Exclusion Criteria	Examples	Crosswalk (Old Constructs Initially Designed Around)
Critical Incidents	NA	critinc	Outer Setting	Discusses large-scale and/or unanticipated events disrupt implementation and/or delivery of heat preparedness and/or response activities	Inclusion: descriptions of heat events, COVID-19, wildfires, and other natural hazards or incidents. May also include discussions of compound and/or cascading events and their impacts during the heat events as well as descriptions of previous disasters (ie wildfires) in relation to response and preparedness activities, such as lessons learned, outstanding capacity issues, or existing networks or protocols that were repurposed.	compound hazards	New with Updated CFIR, rolled compound disasters into this category
Heat Wave Challenges	Critical Incidents	heatchallenge	Outer Setting	Discusses operational, structural, or systemic challenges faced by the healthcare system in providing services during the 2021 heatwave as well as the extreme heat in the summer of 2022	Inclusion: descriptions of challenges attributed to the 2021 heatwave as well as extremely hot days during the summer of 2022. Includes resources that were coveted but not available. e.g. Surges, power loss, etc<br><br>Exclusion: statements about innovations, the success or faults of the heat response, or descriptions of activities associated with the response. These will be coded to Innovation Characteristics and relevant subcodes.	resource scarcity, competing with the public	Added inductively per NE comment, split from Heat Wave Impacts (was made child of Critical Incidents, Updated CFIR construct)
Heat Wave Effects	Critical Incidents	heateffect	Outer Setting	Discusses effects of the heat wave on the community served by the healthcare system and on providers during the 2021 heatwave as well as extreme heat in the summer of 2022	Inclusion: descriptions of effects attributed to the 2021 heatwave as well as extremely hot days during the summer of 2022.<br><br>Exclusion: statements about innovations, the success or faults of the heat response, or descriptions of activities associated with the response. These will be coded to Innovation Characteristics and relevant subcodes.	heat-related illness	Added inductively per NE comment, split from Heat Wave Impacts (was made child of Critical Incidents, Updated CFIR construct)
COVID-19	Critical Incidents	covid	Outer Setting	Discusses challenges and effects of the ongoing COVID-19 pandemic during the 2021 heatwave as well as the extreme heat in the summer of 2022	Inclusion: descriptions or references of COVID-19 or pandemic impacts and related activities.	covid protection measures	Inductive code
Partnerships & Connections	NA	partners	Outer Setting	Discusses the degree to which the organization was networked with other external organizations, including referral networks, academic institutions, other fields/sectors, and coalitions that support heat preparedness and response during the 2021 heatwave as well as the extreme heat in the summer of 2022. This focuses on external relationships.	Inclusion: descriptions of outside group memberships and networking done outside the organization<br><br>Exclusion: statements about general networking, communication, and relationships in the organization such as descriptions of meetings, email groups, or other methods of keeping people connected and informed, and statements related to team formation, quality and functioning (which gets coded to Networks & Communication)		Changed from Cosmopolitanism (old name of this construct), customized description based on NE comment
Recipient-Centeredness	NA	recipcenter	Inner Setting	Discusses perceptions of shared values, beliefs, and norms around treating supporting, and addressing the needs and welfare of those affected by heat	Inclusion: descriptions of organizational beliefs, norms and standards about the targets/participants of heat activities		Changed from Patient Needs & Resources (Old CFIR code)<br><br>Old definition: *The extent to which the needs of those served by the organization, as well as barriers and facilitators to meet those needs, are accurately known and prioritized by the organization.*<br><br>Split across Recipient-Centeredness and Need (New CFIR also split into a third category that we were unsure of, but for our purposes, these captured the main aspects of the old category). Adjusted based on NE comments
Deliverer-Centeredness	NA	delivercenter	Inner Setting	Discusses perceived shared values, beliefs, and norms around treating, supporting, and addressing the needs and welfare of healthcare providers of the heat preparedness and/or response activities	Inclusion: discussions about how the organization prioritizes staff needs, including staff safety		Updated CFIR inductively added
Local Conditions	NA	localcond	Outer Setting	Discusses the economic, environmental, political, and/or technological conditions enabled by the community/service area/local institutions to support implementation and/or delivery of heat preparedness and/or response activities	Inclusion: descriptions of how environmental factors, including social / political / technological factors, support or serve as a barrier for implementation of heat activities<br><br>Exclusion: references to organizational politics, external networks, or interagency relationships	cooling center location, duration of heat	New with Updated CFIR. Added to support replacement of Patient Needs & Resources
Relational Connections	NA	innerrelations	Inner Setting	Discusses the presence of high quality formal and informal relationships, networks, and teams within and across organizational or healthcare sector boundaries (e.g., structural, professional) that are perceived to support or facilitate heat preparedness and/or response. This focuses on internal relationships.	Inclusion: descriptions of organizational relationships within the agency and across agencies, including professional networks that are relied upon to implement heat activities		Old: Networks & Communications. Split into Communications and Relational Connections.<br><br>Old definition: *The nature and quality of webs of social networks, and the nature and quality of formal and informal communications within an organization.*
Available Resources	NA	availresources	Inner Setting	Discusses resources that are or were available to implement and deliver heat preparedness or response activities	Inclusion: statements related to the presence or absence of resources specific to heat preparedness and response activity implementation. Also includes perceptions of resources that were available but not utilized.<br><br>Exclusion: statements related to training and education and code to Access to Knowledge & Information. Does not include resources that were coveted but not available - that codes to challenges.		Old: Readiness for Implementation. This category was removed; available resources is now the parent construct with several other resource-based constructs that we have added.<br><br>Old definition: *The level of resources organizational dedicated for heat preparedness and response implementation and on-going operations including physical space and time*
Funding	Available Resources	funding	Inner Setting	Discusses perceived availability of funding to implement and deliver the heat preparedness and response activities during and leading up to the 2021 heat event and subsequent 2022 heat waves	Inclusion: statements related to the availiabilty of organizational funding for heat-related innovations<br>Exclusion: statements related to the cost of innovations or external financing	covid-19 funding	Updated CFIR inductively added
Space	Available Resources	space	Inner Setting	Discusses of the availability of physical space to implement and deliver heat preparedness and/or response activities	Inclusion: statements related to whether or not there was sufficient physical space to implement heat preparedness and response activities<br>Exclusion: statements related to the physical infrastructure itself	beds	Updated CFIR inductively added
Materials & Equipment	Available Resources	supplies	Inner Setting	Discusses the availability of supplies, materials and equipment used to implement and deliver the heat preparedness and/or response	Inclusion: statements about the availability of supplies to implement heat preparedness and response activities, as well as procuring those supplies	ice, popsicles, portable AC	Updated CFIR inductively added
Access to Knowledge and Information	NA	knowledgeaccess	Inner Setting	Discusses perceived accessibility of guidance and/or training to implement heat preparedness and response activities	Inclusion: statements related to implementation leaders' and users' access to knowledge and information regarding heat preparedness and response plans<br><br>Exclusion: statements related to engagement strategies and outcomes, e.g., how key stakeholders became engaged with the heat preparedness and response plans are and what their role is in implementation, and code to Engaging: Key Stakeholders. Also, statements about general networking, communication, and relationships in the organization, such as descriptions of meetings, email groups, or other methods of keeping people connected and informed, and statements related to team formation, quality, and functioning, and code to Networks & Communications	tip sheets, trusted partner communications	Old: Readiness for Implementation/ Access to Knowledge and Information This category was removed; access to knowledge and information remains its own category<br><br>Old definition: *Ease of access to digestible information and knowledge about the innovation and how to incorporate it into work tasks*

Relative Priority	NA	priority	Inner Setting	Discusses perceptions that implementing and delivering the heat preparedness and response efforts was or is important compared to other initiatives	Inclusion: statements that reflect the relative priority of heat preparedness and response activities, e.g., statements related to change fatigue in the organization due to implementation of many other programs.<br><br>Exclude: statements regarding the priority of the innovation based on compatibility with organizational values		Updated definition per Updated CFIR.<br><br>Old definition: *Individuals' shared perception of the importance of the implementation within the organization.*
Planning	NA	planning	Process	Discusses the presence of planning that identifies roles and responsibilities, outlines specific steps and milestones, and defines goals and measures for implementation success in advance of a heat event	Inclusion: evidence of pre-implementation diagnostic assessments and planning, as well as refinements to the plan. Also includes discussion of planning for activities that could have been but were not utilized, as well as planning that has been adopted since the 2021 or 2022 heat season and lessons learned that have been implemented<br><br>Exclusion: statements about partnerships, external relationships, and/or the implementation of the heat activities	explicit heat action plans	Updated definition per Updated CFIR.<br><br>Old definition: *The degree to which a scheme or method of behavior and tasks for implementing the heat preparedness and/or response activities were developed in advance, and the quality of those schemes or methods.*
Engaging	NA	engaging	Process	Discusses attempts to attract and encourage participation from community members, responders, and other stakeholders in the implementation and/or the design of heat preparedness and response activities during the 2021 event and subsequent 2022 heat waves.	Inclusion: statements related to engagement strategies and outcomes, i.e., if and how staff and individuals who became engaged with people who participated or engaged in the heat activities and what their role is in implementation; also includes how individuals may have been engaged byt were not<br><br>Exclusion: statements about networks, partners, or other formal organizational relationships	outreach to patients via telehealth	Updated definition per Updated CFIR.<br><br>Old definition: *Attracting and involving appropriate individuals in the implementation and use of the heat preparedness and response activities through a combined strategy of social marketing, education, role modeling, training, and other similar activities.*
Reflecting & Evaluating	NA	evaluating	Process	Discusses approaches and data collected (both quantitative and qualitative) (or that could/should have been taken/collected) to assess progress and quality of heat preparedness and response.	Inclusion: statements that refer to the implementation team's assessment activities surrounding the progress toward and impact of implementation, as well as the interpretation of outcomes related to implementation. Reflecting and Evaluating is part of the implementation process; it likely ends when implementation activities end. It does not require goals be explicitly articulated; it can focus on descriptions of the current state with real-time judgment	AARs, hotwashes	Updated definition per Updated CFIR.<br><br>Old definition*: Collect and discuss quantitative and qualitative information about the success of implementation and/or the innovation.Use this construct to capture themes related to Reflecting & Evaluating that are not included in the subconstructs below*
Capability	NA	capability	Individuals: Characteristics	Discusses perceptions that the individual(s) has knowledge, and skills, including interpersonal skills, to fulfill role in heat preparedness and response activities	Inclusion: statments related to whether the individual had or lacked requisite knowledge to implement heat preparedness and response activities; knowledge gaps about the implementation		Old: Self Efficacy. Split into three with removal of old "characteristics" domain- Capability, Opportunity, and Motivation<br><br>Old definition: *Individual belief in their own capabilities to execute courses of action to achieve implementation goals*
Opportunity	NA	opportunity	Individuals: Characteristics	Discusses perceptions that the individual(s) has availability, scope/authority, and power to fulfill role in in heat preparedness and response activities	Inclusion: statements related to whether the individual had access to the available resources and appropriate situational conditions to implement the heat response innovations		Old: Self Efficacy. Split into three with removal of old "characteristics" domain- Capability, Opportunity, and Motivation<br><br>Old definition: *Individual belief in their own capabilities to execute courses of action to achieve implementation goals*
Policies and Laws	NA	policies	Outer Setting	Discusses legislation, regulations, sector-wide policies, professional group guidelines and recommendations, or accreditation standards that support implementation and/or delivery of heat preparedness and response.	Inclusion: Discussion of external policies, practices, guidelines, etc. that impact the availability of heat preparedness and response plans or the delivery of heat preparedness and response activities	DIVERT, level-loading	Updated CFIR inductively added (based on similar construct in old CFIR)
Structural Characteristics	NA	structural	Inner Setting	Discusses infrastructure components that support functional performance of the healthcare organization or sector during extreme heat events	Inclusion: Statements of social or physical structures that impact the implementation or delivery of a heat response innovation<br><br>Exclusion: Discussions of infrastructure included under physical or work infrastructure		Updated CFIR inductively added. Significant departure from related concept in old CFIR
Physical Infrastructure	Structural Characteristics	physinfra	Inner Setting	Discusses the layout and configuration of space and other tangible material features that support functional performance of the healthcare organization or sector	Inclusion: descriptoins of the physical structures and supportive physical infrastructure (energy, fuel, vehicles, etc) that are required to implement heat preparedness and response activities<br><br>Exclusion: references to funding, human resources, or political/social/environmental factors that are part of the outer setting	Generators, ambulances, temperature control, beds	Updated CFIR inductively added
Work Infrastructure	Structural Characteristics	workinfra	Inner Setting	Discusses the organization of tasks and responsibilities within and between individuals and teams, and general staffing levels, that support functional performance of the healthcare organization or sector	Inclusion: descriptions of the human resources that comprise the heat preparedness and response workforce, including discussions of staff shortages, the organization of the workforce, etc<br><br>Exclude: discussions of the capacity of the workforce		Updated CFIR inductively added
Tailoring Strategies	NA	tailorstrat	Process	Discusses the layout and configuration of space and other tangible material features that support functional performance of the healthcare organization or sector during extreme heat events, as well as perceptions of the extent that heat preparedness and response implementation plans and activities can be modified, tailored, or refined to fit local context or needs	Inclusion: discussions of how heat preparedness and response activities are structured in order to most effectively reach the intended audience, including how barriers are overcome. Also includes statements regarding the (in)ability to adapt the innovation to their context, e.g., complaints about the rigidity of the protocol. Suggestions for improvement can be captured in this code, even if it is clear that the participant feels the change is needed but that the program cannot be adapted. Includes both community and organizational adaption of heat response/ preparedness<br><br>Exclusion: statements that heat preparedness and response activities did or did not need to be adapted or that there is room for improvement but adaptation cannot be made		Updated CFIR inductively added
