# Supplementary material for: Lessons learnt from the 2021 Pacific Northwest heat dome: a qualitative study of western Washington’s healthcare community response
Source: BMJ Open. 2025 Apr 17;15(4):e089093. doi: 10.1136/bmjopen-2024-089093 (PMC12007061; doi:10.1136/bmjopen-2024-089093)
Supplement: online supplemental material 5 [file bmjopen-15-4-s005.docx]

## **Supplemental Materials 5: Recommendations for Policy and Practice**

The following table outlines recommendations based on our findings for improving EHE response activity implementation in both policy and practice. In addition to actions that can be taken at any stage, we specifically mention recommendations that can be implemented in the lead-up to an event (preparedness), during an event (response), and after an event (evaluation).

| **All Stages** |
| --- |
| Identify a lead organization for centralized coordination |
| Compile resources and information related to EHE preparedness and response |
| Develop a regional asset awareness/asset sharing system |
| Convene diverse organizations from across the health sector before and after events |
| **Preparedness** |
| Make resilience investments that have co-benefits for heat and other hazards |
| Find existing practices that can be adapted for extreme heat response |
| Conduct outreach early and often |
| Build redundancy into response roles |
| **Response** |
| Incentivize heat-protective behaviors |
| Prioritize creating "cool zones" instead of cooling entire facility if faced with resource pressures |
| Provide supportive spaces and resources for staff during the event |
| Reassess how to manage patient flow between facilities |
| **Evaluation** |
| Codify and institutionalize knowledge and relationships |
| Move towards real-time monitoring and reporting of excess mortality and morbidity |
| Assess EHEs in the context of other climate-sensitive hazards |
