# Supplementary material for: Lessons learnt from the 2021 Pacific Northwest heat dome: a qualitative study of western Washington’s healthcare community response
Source: BMJ Open. 2025 Apr 17;15(4):e089093. doi: 10.1136/bmjopen-2024-089093 (PMC12007061; doi:10.1136/bmjopen-2024-089093)
Supplement: Supplementary file 6 [file bmjopen-2024-089093.reviewer_comments.pdf]

# PEER REVIEW HISTORY

BMJ Open publishes all reviews undertaken for accepted manuscripts. Reviewers are asked to complete a checklist review form and are provided with free text boxes to elaborate on their assessment. These free text comments are reproduced below.

### ARTICLE DETAILS

**Title (Provisional)**

Lessons learned from the 2021 Pacific Northwest heat dome: A qualitative study of western Washington's healthcare community response

**Authors**

Korfmacher, Matias; Hartwell, Cat; Hill, Kelly; Matthews-Trigg, Nathaniel; Hess, Jeremy; Nori-Sarma, Amruta; Wellenius, Gregory; Errett, Nicole

---

### VERSION 1 - REVIEW

---

| | |
|---|---|
| **Reviewer** | **1** |
| **Name** | **Bailie, Ross** |
| **Affiliation** | **The University of Sydney, University Centre for Rural Health** |
| **Date** | **29-Jul-2024** |
| **COI** | **none** |

---

This is an interesting paper that addresses an increasingly important area of research as heat becomes a greater threat through climate change.

The paper is well written and generally methodologically sound. I have several questions and comments for consideration in refining the manuscript:

- pg 3, line 44/45 - the acronym AC should be spelled out.

- pg 4, line 41/42 - it would be helpful to have more information on the types of people who attend the NWHRN meetings. Further to this, are the groupings for the focus groups reflective of the attendees? Were there other types of people included in these listening sessions? Were there other types of people who were not included who could have provided an important perspective? Who/what types of people?

Also - what of perceptions of the public - users and should/would be users of the health system? Are these reflected by participants in the listening sessions and focus groups? This does not seem to be the case; if this is correct it seems to be an important limitation that warrants further comment in the limitations section of the discussion.

- pg 4, line 49/50 - similarly, was the professional affiliation the only consideration in the sampling process? Was consideration given to other factors that might have resulted in biased perspectives, representation of particular interest groups, lack of representation by important groups? The extent of representation of all relevant perspectives warrants further comment in the discussion, including with regard to priorities for further research to ensure missing perspectives are addressed.

- it is not clear from the 'grouping by organisation type' if PHC services/ primary medical services/ family physicians are represented; this should be clarified. Similar to the points made above, this is an issue that warrants further comment in the discussion.

- pg 5, line 25 - the use of the CFIR is an interesting choice for this study as the aim/research question is not clearly about implementation of a specific intervention or program. It would be useful to have a clear justification for use of the CFIR in this analysis, as well as an assessment of the extent to which the CFIR was considered fit-for-purpose; i.e. did the data fit neatly into this framework - were there data/themes that did not fit well? Where there components of the framework that could have been adjusted to provide a framework that is more reflective of the scope and range of themes emerging from the data? What are the implications of this for further research?

- page 6, lines 45/47 - The sentence 'Responses to the heat dome differed depending on local conditions, such as jurisdictional differences between emergency response agencies, rural versus urban setting, organizational resources, and the perceived level of public trust in the healthcare system' seems important. However, the differences in responses in relation to these 'local conditions' are not teased out well in further presentation of the results or the discussion. This seems important with regard to

the implications of the findings for policy and practice.

- page 9 first paragraph states 'Few reported having heat-specific response plans at the time, though some mentioned adapting emergency response and other hazard-related plans (such as mass casualty event plans) or creating staffing and treatment plans immediately before the event. Several indicated that such plans improved their heat responses.' There does not appear to be any reference in the results or the discussion to an evidence base on which responses could/should be based, or the availability of evidence-based guidelines for health service/health system responses to EHEs. Did this not emerge as an issue? If not, were the authors surprised at the lack of reference to an evidence base or to relevant evidence-based guidelines? Are the authors aware of such an evidence base or guidelines? What are the implications of this for policy, practice and future research? These seem important issues to be addressed in the discussion.

- Figure 1 presents 'Reported activities and responsibilities by healthcare sector'' it is not clear where primary health care and primary medical care fit in this typology of organisations that make up the health sector? And where do research organisations fit? do they have a role? e.g. reviews of the evidence on effective interventions/responses,

producing evidence-based guidelines, conducting primary research to address gaps in the evidence? The role of health research institutions is relevant to the above comments about the evidence base and guidelines.

---

| | |
|---|---|
| **Reviewer** | **2** |
| **Name** | **Houghton, Adele** |
| **Affiliation** | **Biositu** |
| **Date** | **21-Aug-2024** |
| **COI** | **None** |

---

General Comments

This article shares results from a comprehensive qualitative analysis of healthcare system response in western Washington to the 2021 heat dome event. The article is well written and well organized with a strong methods section and clearly articulated results. It adds a significant contribution to the literature on health system preparedness for climate change-related events. The figures and tables in particular offer specific information that could be implemented by health system emergency management and climate resilience programs.

Specific Comments

[Note: All page numbers below refer to the "Page X of 33" page number listed on the top righthand corner of the manuscript.]

Introduction - General: Two key challenges to health system EHE preparedness are missing from the section: 1) the fact that the definition of an extreme heat event changes from one place to the next (which can cause confusion); and, 2) the fact that EHEs are currently not defined as "disasters" by FEMA (which can complicate activating EHE protocols, emergency management operations, access to disaster funding mechanisms, etc.).

p. 4, lines 3-7: Two additional common heat preparedness activities are weatherization programs and fan/window air conditioning unit distribution programs.

p. 4, lines 9-14: Arguably the most significant impact of EHEs on the health sector is their tendency to cause brownouts and blackouts, which directly impact facility operations as well as exposing at risk groups to dangerously hot indoor air temperatures. I see you mention this stressor on page 11 (lines 10-14). I would suggest either moving that sentence to the introduction or referencing it twice.

Results – General. The results section would be strengthened by adding more quotes and specifics. For example, Section III.1. (p. 7, lines 29-34) lump together a wide range of topics that would be more helpful to readers if they were described in more detail.

## VERSION 1 - AUTHOR RESPONSE

### Reviewer: 1

*Comments to the Author:*

This is an interesting paper that addresses an increasingly important area of research as heat becomes a greater threat through climate change. The paper is well written and generally methodologically sound. I have several questions and comments for consideration in refining the manuscript.

**Thank you for your generous support and for taking the time to review our manuscript. We have responded to each of your comments and those of Reviewer 2 below, and made associated changes as outlined. We believe these changes have improved the manuscript, and very much appreciate your expertise and perspective.**

pg 3, line 44/45 - the acronym AC should be spelled out.

**Thank you for your comment. We have made this change and applied AC for consistency throughout the manuscript.**

pg 4, line 41/42 - it would be helpful to have more information on the types of people who attend the NWHRN meetings. Further to this, are the groupings for the focus groups reflective of the attendees? Were there other types of people included in these listening sessions? Were there other types of people who were not included who could have provided an important perspective? Who/what types of people?

**Thank you for your comment. We have incorporated responses to your questions into a methods supplement detailing the focus group selection process, the pool of participants from the listening sessions, and their roles within their respective organizations. This information can be found in Supplemental Materials 2.**

Also - what of perceptions of the public - users and should/would be users of the health system? Are these reflected by participants in the listening sessions and focus groups? This does not seem to be the case; if this is correct it seems to be an important limitation that warrants further comment in the limitations section of the discussion.

**Thank you for your comment. We have made note of the absence of the public's perspective in limitations, and would like to clarify that this project was primarily focused on the perspectives of those implementing response activities throughout the sector, rather than on the discrete impacts of the heat dome itself.**

pg 4, line 49/50 - similarly, was the professional affiliation the only consideration in the sampling process? Was consideration given to other factors that might have resulted in biased perspectives, representation of particular interest groups, lack of representation by important groups? The extent of representation of all relevant perspectives warrants further

comment in the discussion, including with regard to priorities for further research to ensure missing perspectives are addressed.

**Thank you for your comment. We have added additional context to the methods and limitations sections, as well as to Supplemental Materials 2 to address these comments about the selection process and participant perspectives.**

it is not clear from the 'grouping by organisation type' if PHC services/ primary medical services/ family physicians are represented; this should be clarified. Similar to the points made above, this is an issue that warrants further comment in the discussion.

**Thank you for the opportunity to clarify our language. We have added a methods supplement (Supplemental Materials 2) and additional language to add context to the perspectives represented. As a note, we were operating from the perspective that primary health care and primary medical care are broad organizational responsibilities; each is covered by several organization types within our focus group categories.**

pg 5, line 25 - the use of the CFIR is an interesting choice for this study as the aim/research question is not clearly about implementation of a specific intervention or program. It would be useful to have a clear justification for use of the CFIR in this analysis, as well as an assessment of the extent to which the CFIR was considered fit-for-purpose; i.e. did the data fit neatly into this framework - were there data/themes that did not fit well? Where there components of the framework that could have been adjusted to provide a framework that is more reflective of the scope and range of themes emerging from the data? What are the implications of this for further research?

**Thank you for your comment. Implementation science has historically been broadly concerned with closing the gap between the body of evidence in health research with actual practice in healthcare delivery. In the context of this research, “extreme heat event (EHE) response activities” are interpreted as a suite of interventions that practitioners, based on author experiences and perspectives expressed during the listening sessions, tend to view in aggregate. There is also the practical matter of assessing a wide range of activities across the whole health sector; the individual activities that make up an organization’s total heat response vary based on the role the implementing organization plays in the health sector, and may even vary between organizations serving the same function due to local circumstances. By taking a sector-wide approach, this research seeks to identify the system-level barriers, facilitators, and lessons learned related to EHE response activities that may not be otherwise evident. By interrogating how a region unaccustomed to EHEs actually goes about implementing effective response, our findings serve as an important evidence base for improving the whole health sector’s response capabilities. Ultimately, the authors felt that the breadth of the Updated CFIR constructs and the ability to select those most relevant felt flexible and responsive to the study’s context. We have added an explanation of our use of CFIR alongside a table reflecting on the justification for each construct's selection, as well as an overall justification of the use of the Updated CFIR framework and our interpretation of this study's contribution to the**

**larger body of implementation science research. You can find this as Supplemental Materials 1. It is important to note that this study's research questions were not explicitly aimed at evaluating the CFIR's fit to the setting; the authors have included this supplementary materials to reflect on the fit of the framework to the project and to encouragement to explore the use of the CFIR in innovative contexts (e.g., on other hazard-specific approaches to climate adaptation).**

page 6, lines 45/47 - The sentence 'Responses to the heat dome differed depending on local conditions, such as jurisdictional differences between emergency response agencies, rural versus urban setting, organizational resources, and the perceived level of public trust in the healthcare system' seems important. However, the differences in responses in relation to these 'local conditions' are not teased out well in further presentation of the results or the discussion. This seems important with regard to the implications of the findings for policy and practice.

**Thank you for your comment. We agree that better understanding how various facets of local conditions shape extreme heat response has important implications for policy and practice, and have added language acknowledging it as an important direction for future research. However, this project did not present a systematic investigation of specific differences by jurisdictional location. Rather, we are reporting shared experiences where applicable; the variation by location is noted as an important background detail rather than as a key research focus. Local variations are acknowledged most explicitly in the results section in the subheading "Tailoring Strategies to Local Context" (formerly "Tailoring Strategies to Patient/Community Preferences"), and we have added additional signposting language to that section.**

page 9 first paragraph states 'Few reported having heat-specific response plans at the time, though some mentioned adapting emergency response and other hazard-related plans (such as mass casualty event plans) or creating staffing and treatment plans immediately before the event. Several indicated that such plans improved their heat responses.' There does not appear to be any reference in the results or the discussion to an evidence base on which responses could/should be based, or the availability of evidence-based guidelines for health service/health system responses to EHEs. Did this not emerge as an issue? If not, were the authors surprised at the lack of reference to an evidence base or to relevant evidence-based guidelines? Are the authors aware of such an evidence base or guidelines? What are the implications of this for policy, practice and future research? These seem important issues to be addressed in the discussion.

**Thank you for your comment. During our focus groups we did not hear reference to which, if any, evidence-based guidelines were or were not available for making plans. While participants did not make explicit reference to any evidence-based guidelines in relation to creating their plans or informing their response strategies, they did reference several important knowledge resources (particularly those obtained through relationships and partnerships with other organizations) that influenced their organization's activities. Our focus group facilitation questions did not investigate the evidence base for these knowledge resources; as such, our data does not support**

**making an assertion about the extent to which evidence-based guidelines were incorporated into heat action planning and response. Rather, it is noteworthy that while we note in the introduction that evidence-informed interventions have been identified but not widely implemented, many participants reported that these interventions were part of their response strategies. Given that the heat dome was unprecedented for participants, reflected in the "building the plane while flying it" illustrative quote, while they concurrently described integration of strategies known to be supported by evidence, suggests that there was at least some regional awareness of evidence-based strategies and interventions. We have added a line to the discussion reflecting that our findings suggest such awareness, as well as clarifying language to the results section and Table 2.**

Figure 1 presents 'Reported activities and responsibilities by healthcare sector'' it is not clear where primary health care and primary medical care fit in this typology of organisations that make up the health sector? And where do research organisations fit? do they have a role? e.g. reviews of the evidence on effective interventions/responses, producing evidence-based guidelines, conducting primary research to address gaps in the evidence? The role of health research institutions is relevant to the above comments about the evidence base and guidelines.

**Thank you for the opportunity to clarify our methods. We have addressed this both through a comment in limitations and in the Supplemental Materials 2. Primary health care and primary medical care we viewed as cross-cutting responsibilities that are covered by several organization types within our focus group categories. We engaged primarily with NWHRN's emergency manager contact at each organization; given the response-sided focus of this paper, that did not include research organizations, as they did not play an active role in implementing the response activities themselves.**

## Reviewer: 2

*Comments to the Author:*
<u>General Comments</u>
This article shares results from a comprehensive qualitative analysis of healthcare system response in western Washington to the 2021 heat dome event. The article is well written and well organized with a strong methods section and clearly articulated results. It adds a significant contribution to the literature on health system preparedness for climate change-related events. The figures and tables in particular offer specific information that could be implemented by health system emergency management and climate resilience programs.

**Thank you for your kind encouragement and for taking the time to review our manuscript. We have responded to both your comments and those of Reviewer 1 below, and made associated changes as outlined. We believe these changes have improved the manuscript, and very much appreciate your expertise and perspective.**

Specific Comments
[Note: All page numbers below refer to the “Page X of 33” page number listed on the top righthand corner of the manuscript.]

Introduction - General: Two key challenges to health system EHE preparedness are missing from the section: 1) the fact that the definition of an extreme heat event changes from one place to the next (which can cause confusion); and, 2) the fact that EHEs are currently not defined as “disasters” by FEMA (which can complicate activating EHE protocols, emergency management operations, access to disaster funding mechanisms, etc.).

**Thank you for your comment. We have revised text in the introduction that acknowledges both challenges at a high level (particularly noting the lack of major disaster declarations under the Stafford Act), and have incorporated additional references to support these assertions.**

p. 4, lines 3-7: Two additional common heat preparedness activities are weatherization programs and fan/window air conditioning unit distribution programs.

**Thank you for your comment. We have added language referencing the use of personal fan/window air conditioner distribution programs as common practice in the sector (which is also cited in Randazza et al). However, we have opted to omit language about weatherization; while acknowledging the importance of such measures in protecting population health, weatherization programs are typically carried out by housing or energy agencies, which fall beyond the scope of the health sector as defined in this paper.**

p. 4, lines 9-14: Arguably the most significant impact of EHEs on the health sector is their tendency to cause brownouts and blackouts, which directly impact facility operations as well as exposing at risk groups to dangerously hot indoor air temperatures. I see you mention this stressor on page 11 (lines 10-14). I would suggest either moving that sentence to the introduction or referencing it twice.

**Thank you for this comment. We have added language specifically acknowledging power supply challenges earlier in the introduction, and have incorporated additional references both in the introduction and in the discussion to reflect the magnitude of the issue.**

Results – General. The results section would be strengthened by adding more quotes and specifics. For example, Section III.1. (p. 7, lines 29-34) lump together a wide range of topics that would be more helpful to readers if they were described in more detail.

**Thank you for your comment. While we agree that additional pull quotes and specific examples would provide additional details, after balancing their addition with the other substantive content revisions that you and the other reviewer suggested, we have had**

**to make the hard decision of omitting illustrative quotes to respect the word count limitations of the journal.**
